# Should LSIL with ASC-H (LSIL-H) in cervical smears be an independent category? A study on SurePath™ specimens with review of literature

**DOI:** 10.1186/1742-6413-4-7

**Published:** 2007-03-20

**Authors:** Vinod B Shidham, Nidhi Kumar, Raj Narayan, Gregory L Brotzman

**Affiliations:** 1 Department of Pathology, Medical College of Wisconsin, Milwaukee, Wisconsin, USA; 2 Obstetrics and Gynecology, Medical College of Wisconsin, Milwaukee, Wisconsin, USA; 3 Family and Community Medicine, Medical College of Wisconsin, Milwaukee, Wisconsin, USA

## Abstract

**Background:**

Cervical smears exhibiting unequivocal features of 'low grade squamous intraepithelial lesion' (LSIL) are occasionally also admixed with some cells suspicious for, but not diagnostic of, 'high grade squamous intraepithelial lesion' (HSIL). Only a few studies, mostly reported as abstracts, have evaluated this concurrence. In this study, we evaluate the current evidence that favors a distinct category for "LSIL, cannot exclude HSIL" (LSIL-H), and suggest a management algorithm based on combinations of current ASCCP guidelines for related interpretations.

**Methods:**

We studied SurePath™ preparations of cervical specimens from various institutions during one year period. Cytohisto correlation was performed in cases with cervical biopsies submitted to our institution. The status of HPV DNA testing was also noted in some LSIL-H cases with biopsy results.

**Results:**

Out of 77,979 cases 1,970 interpreted as LSIL (1,523), LSIL-H (146), 'atypical squamous cells, cannot exclude HSIL' (ASC-H) (109), and HSIL (192) were selected. Concurrent biopsy results were available in 40% (Total 792 cases: 557 LSIL, 88 LSIL-H, 38 ASCH, and 109 HSIL). Biopsy results were grouped into **A**. negative for dysplasia (ND), **B**. low grade (HPV, CIN1, CIN1 with HPV), and **C**. high grade (CIN 2 and above).

The positive predictive values for various biopsy results in relation to initial cytopathologic interpretation were: **a**. LSIL: (557 cases): ND 32% (179), low grade- 58% (323), high grade- 10% (55); **b**. LSIL-H: (88 cases): ND 24% (21), low grade- 43% (38), high grade- 33% (29); **c**. ASCH: (38 cases): ND 32% (12), low grade- 37% (14), high grade- 31% (12); **d**. HSIL (109 cases): ND 5% (6), low grade 26% (28), high grade 69% (75). The patterns of cervical biopsy results in cases reported as LSIL-H were compared with that observed in cases with LSIL, ASC-H, and HSIL.

94% (32 of 34) of LSIL-H were positive for high risk (HR) HPV, 1 was negative for HR HPV but positive for low risk (LR), and 1 LSIL-H was negative for HR and LR both.

**Conclusion:**

LSIL-H overlapped with LSIL and ASC-H, but was distinct from HSIL. A management algorithm comparable to ASC-H and HSIL appears to be appropriate in LSIL-H cases.

## Background

Cervical smears may exhibit unequivocal 'low grade squamous intraepithelial lesion' (LSIL) in association with atypical cells cytomorphologically suspicious for, but not sufficient to be interpreted as, 'high grade squamous intraepithelial lesion' (HSIL) [1,2]. This concurrence has been identified recently by many laboratories, but its reporting is not addressed in the 2001 Bethesda System terminology (Bethesda 2001) [3-5]. Lack of a standardized method of reporting, however, may affect proper application of American Society for Colposcopy and Cervical Pathology (ASCCP) guidelines based on Bethesda 2001 [6,7]. Although 'atypical squamous cells, cannot exclude HSIL' (ASC-H) is not a definitive interpretation, it is related to an increased risk of higher-grade lesions on biopsy [3]. On the contrary, LSIL is a definitive interpretation with relatively lower prevalence of high-grade lesion on subsequent biopsy.

Currently, the reporting pattern to communicate this concurrence varies amongst different cytopathology laboratories. In our institution, for statistical and quality assurance reasons, we report this concurrence under definitive interpretation as LSIL with a comment that ASC-H cells are also present. Others essentially follow a reverse approach and report it as ASC-H with LSIL in the comment. Some interpreters may choose to combine the associated ASC-H component with LSIL and report the combination as HSIL, which may lead to a potentially high false positivity rate. Rarely, the ASC-H component may be downgraded to 'atypical squamous cells of undetermined significance' (ASCUS) with the final interpretation as LSIL, with potentially false negative results for high-grade lesions.

**Figure 2:**
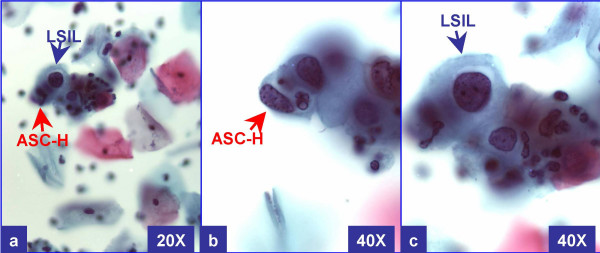
**LSIL-H (with CIN2 & HPV in biopsy)**: Cervical smear with unequivocal LSIL in other fields. This field shows rare LSIL (a & c) with some groups of cells consistent with ASC-H. The cells have a high N/C ratio with rounder curving cell borders (better seen in 'b'). *At 20X (a), the ASC-H cell is difficult to focus because of three dimensional component in liquid based cytology*. (a through c- Papanicolaou stained SurePathTM preps)

**Figure 3:**
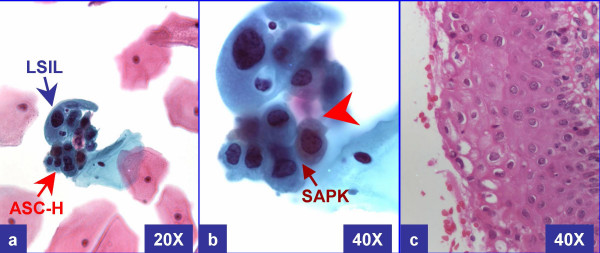
**LSIL-H (with only HPV in biopsy)**: Cervical smear (a, b) showed unequivocal LSIL cells in other fields. This field shows rare LSIL (a & b) along with some groups of cells consistent with ASC-H. The biopsy (c) showed only human papilloma virus cytopathic effect. Small atypical parakeratotic (SAPK) cells with distinct and sharp angulated cell borders with tinge of cytoplasmic eosinophilia (arrowhead in b) (see also corresponding area in a) were interpreted as ASC-H component. (a & b- Papanicolaou stained SurePathTM preps, c- HE stained cervical biopsy section).

In the current study, we evaluated cervical biopsies in cases of "LSIL with ASC-H" (LSIL-H) (Figure 2 &3) in comparison to other categories in Bethesda 2001, to see if this designation as a distinct category is justified.

### Materials and methods

**Figure 1:**
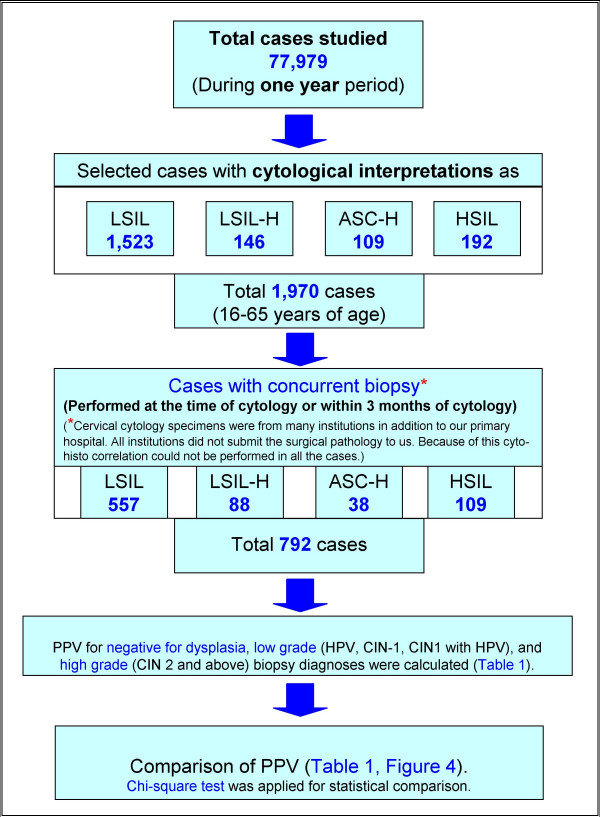
Study plan.

We studied SurePath™ [TriPath Imaging Inc, Burlington, NC] preparations of cervical specimens over a one year period (Figure 1). Initial cytopathologic interpretations were performed by more than five different cytopathologists, based on Bethesda 2001 criteria and were representative of general cytology reporting patterns [4].

LSIL-H was defined as cases with unequivocal LSIL, in concurrence with ASC-H (Figure 2 and 3). ASC-H cells showed cytomorphologic features reported previously [8]. As observed in a subset of ASC-H, some of these cases showed small atypical parakeratotic (SAPK) cells (Figure 3), which may demonstrate superficial resemblance to high grade cells in liquid based cytology [9,10].

The cytology results were correlated with concurrent biopsies (both colposcopically-guided and LEEP biopsies) performed at the time of, or within 3 months of cytologic interpretation. Many institutions, in addition to our primary hospital, submitted cervical cytology specimens. Cervical biopsies from some outside institutions were not sent to our institution for surgical pathology and the cyto-histo correlation was available in only a fraction of cases. The cervical biopsy results for cases with cytologic interpretation as LSIL, LSIL-H, and ASC-H were compared using the Chi-square test for statistical analysis.

In addition, the status of HPV DNA testing (Hybrid Capture (HC) II, Digene, Silver Spring, MD, USA) performed within a year of biopsy was noted in LSIL-H cases with biopsy results.

## Results

We studied 77,979 cases over a one year period (Figure 1). Out of these 1,970 cases (ages 16 to 65 years) were interpreted as abnormal (1,523 LSIL, 146 LSIL-H (Figure 2 &3), 109 ASC-H, and 192 HSIL). The cyto-histo correlation was available in 40% (792 of 1,970) cases. These included 557 LSIL, 88 LSIL-H, 38 ASC-H, and 109 HSIL (Figure 1).

The biopsy results were grouped into: A. negative for dysplasia (ND), B. low grade (HPV, CIN1, or CIN1 with HPV), and C. high grade (CIN 2 and above) (Table 1). Positive predictive value (PPV) for each category of biopsy result was calculated for LSIL, LSIL-H, ASC-H, and HSIL interpretations (Table 1).

**Table 1:** Biopsy results for LSIL, LSIL-H, ASC-H, and HSIL

**Cytopathologic interpretation in SurePath**™		**Positive predictive value (PPV) for**			
		
		**Group A** **Negative result on biopsy **	**Group B** **Low grade (HPV & CIN-1) result on biopsy**	**Group C** **High grade (CIN-2 & CIN-3) result on biopsy **	**Total (792)**
**1**	LSIL*	32% (179/557)	58% (323/557)	**10% **^b^**(55/557)**	100% (557)
**2**	LSIL-H*	**24%**^a ^**(21/88)**	*43%*^c ^(38/88)	33%^b ^(29/88)	100% (88)
**3**	ASC-H*	32%^a ^(12/38)	*37% *^c ^(14/38)	31%^b ^(12/38)	100% (38)
**4**	**HSIL**	5% (6/109)	26% (28/109)	69% (75/109)	100% (109)

* Positive predictive values between groups 1,2,&3 were compared by performing separate 2 × 3 Chi-square tests for rows 1 & 2 and for rows 2 & 3. Applying the Bonferroni correction for multiple testing the significance level was adjusted to 0.025. The statistical comparison between LSIL and LSIL-H shows Chi-square value of 35.7 with p value less than 0.001, consistent with statistically significant difference between these two groups indicating they represent two separate entities. However, for LSIL-H and ASC-H the Chi-square is 0.87 with p value nearly equal to 1, consistent with lack of any significant difference between these two groups.

LSIL-H^b ^and ASC-H^b ^showed higher PPV for high grade dysplasia on biopsy, but prevalence of negative results was lower for LSIL-H, as compared to ASC-H^a^. In summary, LSIL-H overlapped on one side with ASC-H for high grade risk, and with LSIL on other side for higher risk for low grade lesion^c^. The possibility of negative result with LSIL-H^a ^was intermediate between HSIL and LSIL or ASC-H.

**Figure 4:**
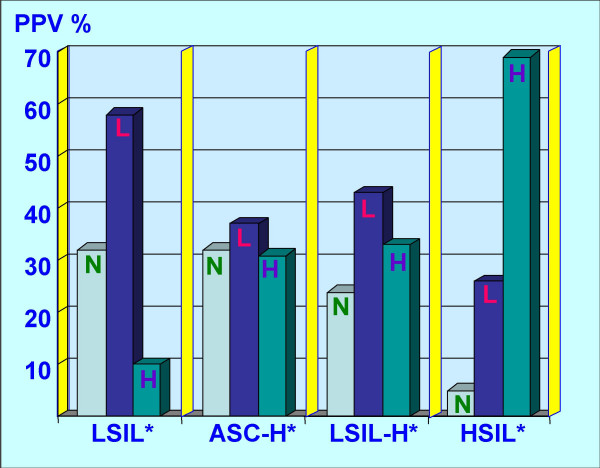
**LSIL-H as category overlap with LSIL and ASC-H, but was distinct from HSIL**. As compared to the LSIL interpretations; LSIL-H and ASC-H showed greater association with high grade dysplasia on biopsy. Compared to LSIL and ASC-H; incidence of negative biopsy results was lower with LSIL-H. However, as compared to HSIL group, LSIL-H had higher incidence of negative results. LSIL-H had higher association with low grade lesion than ASC-H.

LSIL-H (Figure 2 &3) had a lower prevalence of negative biopsy results, compared to ASC-H and LSIL (24% negative results with LSIL-H versus 32% with ASC-H and 32% with LSIL) (Table 1). LSIL-H had a higher chance of association with high grade dysplasia on biopsy, comparable to that for ASC-H (Positive predictive value [PPV] of 33% with LSIL-H and 31% with ASC-H). LSIL alone was associated with a significantly lower risk (PPV 10%) for high grade dysplasia as compared to LSIL-H (PPV 33%). PPV of LSIL-H (33%) was lower for high grade lesions as compared to HSIL (69%). LSIL-H was associated with higher number of negative biopsy results (24%) as compared to HSIL (5%). However, as compared to ASC-H (32%), the prevalence of negative biopsy results with LSIL-H was relatively lower (24%) (Table 1, Figure 4).

PPVs between different groups were compared by performing two separate 2 × 3 Chi-square tests for LSIL versus LSIL-H and LSIL-H versus ASC-H (Table 1). Applying the Bonferroni correction for multiple testing, the significance level was adjusted to 0.025. The differences between LSIL and LSIL-H were statistically significant (Chi-square value of 35.7 with p value less than 0.001). However, for LSIL-H and ASC-H the difference was not significant (Chi-square is 0.87 with p value nearly equal to 1) (Table 1). Thus, the pattern of biopsy results for LSIL-H overlapped on the lower side with LSIL, but was statistically different. On the higher side, it approached ASC-H, without a statistically significant difference, but slightly higher prevalence of negative results as compared to LSIL-H.

Results of HPV DNA testing (HPVT) performed within one year of the concurrent cervical biopsy were available in 34 of 88 LSIL-H cases. 94% (32 out of 34) of LSIL-H cases were positive for high risk HPV. Two cases were negative for high risk HPV. One was negative for both high and low risk HPV, but the biopsy showed mild dysplasia with HPV. The other case was positive for low risk HPV only, but the biopsy showed high grade dysplasia- (not graded).

## Discussion

Lack of a standardized method for reporting LSIL-H generates some questions because of the risk of compromising quality assurance program. More significantly, the proper application of current management guidelines is predominantly based on Bethesda 2001 [6,7] which does not have LSIL-H category. Accumulating evidence, based on our study and a few other studies [1,2,11-14], favor LSIL-H (Figure 2 &3) as a distinct category to address these issues. In the current study, LSIL-H accounted for 0.19% (146 out of 77,979) of all Pap tests. This rate was comparable to that reported by Elsheikh et al (0.15%) [14], Booth et al. (0.15%) [3], and McGrath et al (0.2%) [12].

LSIL-H (Figure 2 &3) had an increased risk of high grade dysplasia on biopsy, which was comparable with ASC-H (33% and 31% respectively), but less than that for HSIL (Table 1, Figure 4). This group, however, had a lower chance of a negative biopsy result as compared to the ASC-H group (24% in LSIL-H versus 32% in ASC-H). As compared to LSIL-H, LSIL alone was associated with a significantly lower risk for high grade dysplasia (33% in LSIL-H versus 10% in LSIL). Thus, LSIL-H cytology showed an increased risk of high grade dysplasia on biopsy, with a PPV higher than that for LSIL cytology, but comparable with ASC-H, and distinctly lower than that for HSIL cytology (Table 1, Figure 4). This confirms the intermediate status of LSIL-H, which overlaps on one side with LSIL, and on other side with ASC-H (Figure 4).

Review of the literature (Table 2) also shows a higher predictive value of LSIL-H for a high grade lesion on biopsy, with overlap on one side with LSIL and on the other with ASC-H, but is distinct from HSIL [1,2,11-13,15-18]. However, most of these studies are reported as abstracts [11,13,15-18] with availability of only partial data for analytical review (Table 2). There are no reports on the use of SurePath liquid based cytology for diagnosis of LSIL-H as reported by this study. Other studies are based on conventional cervical smears or other liquid based cytology such as ThinPrep (Table 2).

**Table 2:** Pattern of results in follow up biopsies- comparative review of literature

**Study**		**Cytology method used in the study**	**Total number of cases studied**	**Number of cases with biopsy**				**High grade dysplasia (CIN2 & above) on biopsy**			
					
				**LSIL**	**LSIL-H**	**ASC-H**	**HSIL**	**LSIL % (n)**	**LSIL-H % (n)**	**ASC-H % (n)**	**HSIL % (n)**
**1**	**Current study [11]**	SurePath™^¶^	77,979 LSIL-H 146 (0.19%) (12 months)	557	88	38	109	10% (55)	33% (29)	31% (12)	69% (75)
**2**	Nasser et al 2003 [1]	Not stated	Not stated (12 months)	150	144	X	X	15% (23)	29% (42)	X	X
**3**	Kir et al 2004 [2]	Not stated	21,342 (2 year)	27	13	X	X	11% (3)	61% (8)	X	X
**4**	McGrath et al ^§ ^2000 [12]	Not stated	48,687 LSIL- 108 (0.2%) (14.5 months)	X	58	X	X	X	59% (34)	X	X
**5**	Elsheikh et al 2006 [14]	ThinPrep^®^*	129,911 LSIL- 194 (015%) (25 months)	575	59	110	289	13% (75)	41% (24)	45% (49)	74% (214)
**6**	Booth et al 2005 [13] (**Abstract**)	Not stated	21,082 LSIL-H 31 (0.15%)	X	X	X	X	10% (5)	45% (9)	X	69% (29)
**7**	D'Furio et al 2005 [17] (Abstract)	ThinPrep^®^*	Not stated	X	83	37	X	X	40%	62%	X
**8**	Underwood et al 2006 [15] (**Abstract**)	ThinPrep^®^&Conventional	130,761 **A. **ThinPrep^®^* (127,929) **B. **Conventional (2832) LSIL-H 270 (0.2%) (24 months)	X	X	X	X	13% (163)	36% (70)	38% (93)	66% (170)
**9**	O'Brien et al 2006 [16] (**Abstract**)	ThinPrep^®^*	**A. ***Pre-imaging*: 76,365 (50 LSIL-H 0.065%) **B. ***Post-imaging*:63,812 (139 LSIL-H 0.22%)	X	A- 40 B- 107	X	X	X	A- 23%(9) B- 37%(39)	X	X
**10**	Jain et al 2005 [18] (**Abstract**)	Not stated	Total 67 LSIL-H **A**- *Few *: **3 or more **ASC-H cells **B**- *Rare*: **1 to 2 **ASC-H cells	X	48 A. 22 B. 26	X	X	X	A- 64% (14/22) B- 23% (6/26)	X	56% (9/16)

^§^This study used different terminology as mild to moderate dysplasia but implied non-definitive interpretation equivalent to LSIL-H; ^¶^SurePath™ ((TriPath Imaging, Inc. Burlington, NC, USA)), *

ThinPrep^® ^(Cytyc Corporation, Marlborough, MA, USA), m, months; yrs, years.

X Blanks represent lack of that information in the corresponding published data.

The abstract reported by Booth et al showed association of LSIL-H cytologic interpretation with high grade dysplasia in 45% of cases on biopsy, as compared to 10% in the LSIL group [13]. These researchers evaluated LSIL-H with reference to ThinPrep imaging [16]. Their abstract showed post-imaging increases in the interpretation of LSIL-H, with a higher predictive value as compared to pre-imaging figures (64% versus 23% with statistically significant difference) [16]. Another abstract by Jain et al reported the significance of number of ASC-H cells in association with LSIL, with the conclusion that the PPV for high grade dysplasia on biopsy increases with higher numbers of ASC-H cells in the smear [18]. Additional abstracts reported variable overlap of LSIL-H with ASC-H and LSIL [15,17].

McGrath et al reported 58 cases initially interpreted as mild to moderate dysplasia in conventional cervical smears, with follow-up biopsies showing high grade dysplasia in 59% and low grade dysplasia in 41% [12]. However, this study did not use the current LSIL-H terminology. Based on univariate and multivariate logistic regression analysis, they did not find any specific morphologic features or relationship with the volume of LSIL-HSIL components for definitive interpretation. Nasser et al used a slightly different approach than our study, and compared 144 LSIL-H cases with 155 LSIL cases (average follow-up, 3–4 months) [12]. They reported a higher incidence of high grade dysplasia on biopsy in cases with LSIL-H as compared to LSIL cytology (29% for LSIL-H vs.15% for LSIL). Out of 21,342 cases evaluated by Kir et al, 13 LSIL-H interpretations were associated with high grade dysplasia on biopsy in 61% of cases, as compared to only 11% high grade dysplasia on biopsy in 27 cases with LSIL cytology [2]. A study by Elsheikh et al [14] evaluated LSIL-H in ThinPrep. All these studies recommended LSIL-H as a distinct cytologic diagnosis (Table 1). Our results with SurePath are comparable to those reported by Elsheikh et al for ThinPrep [14] and Underwood et al [15] for conventional smears and ThinPrep.

A subset of LSIL-H is associated with HPV, CIN1 or CIN1 with HPV on biopsy (Figure 4). As observed in a subset of ASC-H, some of these cases may be unequivocal cases of LSIL with small atypical parakeratotic (SAPK) cells (Figure 3) which may demonstrate a superficial resemblance to high grade cells in liquid based cytology [9,10,19]. Careful scrutiny with reference to the morphological spectrum of ASC-H reported previously should facilitate improved interpretation even with cytomorphology [8]. Some of the ASC-H patterns may represent hyperchromatic crowded groups which should be scrutinized carefully for proper interpretation as reported previously [21,22].

**Figure 5:**
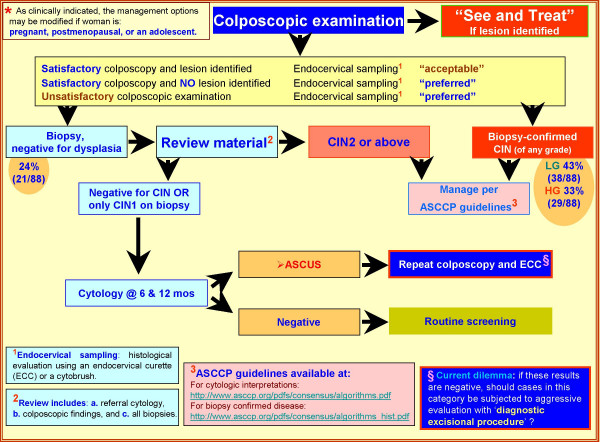
Suggested Management algorithm of Women with LSIL-H*. §Diagnostic excisional procedure- Sampling of transformation zone and endocervical canal for histological evaluation with laser conization, cold-knife conization, loop electrosurgical excision (LEEP), and loop electrosurgical conization.

The management of LSIL-H cases has not been addressed currently by the ASCCP guidelines [6,22-25]. Based on the biopsy pattern in this study and review of the literature, the initial management may be similar to that of LSIL with referral to colposcopy [6,7], but the subsequent approach may be comparable to HSIL (and ASC-H) (Figure 5). Or, the management may be entirely similar to ASC-H. Some of the issues to be considered while planning management guidelines include: A. How to manage patients with negative or unsatisfactory colposcopy results? B. Would a conservative approach similar to LSIL or ASC-H cases be optimum? C. Should endocervical sampling be obtained if the colposcopic examination is satisfactory? D. Would a cone biopsy similar to HSIL cases ever be indicated in evaluation of LSIL-H category?

As the risk of high grade dysplasia associated with LSIL-H is comparable with ASC-H, (Table 1, Figure 4) and the association with lower incidence of negative results is similar to LSIL, it is reasonable to apply some combination of ASC-H and LSIL management guidelines. Possible application of the HSIL approach at later stages of the algorithm may be needed (Figure 5). For ASC-H and LSIL, ASCCP recommends initial colposcopy [6,26]. If the colposcopic evaluation in the LSIL-H management algorithm suggested in Figure 4 is negative, the cytology and biopsy material should be reviewed. If the review leads to a change in the interpretation, one should follow the appropriate ASCCP algorithm [6,26]. As compared to the choice of following either the LSIL or ASC-H algorithm, the application of the suggested guidelines will require a relatively small proportion (approximately 1 out of four cases) of already rare LSIL-H cases to go through slightly increased number of clinical encounters (Figure 5). These guidelines may be evaluated comparatively with other possible alternative combinations to refine it further as indicated by follow up studies in the future.

Another alternative to LSIL-H as a distinct category is to continue with current approach of communicating two distinct interpretations, LSIL and ASC-H, for a given single cervical specimen. Although this approach accommodates 2001 Bethesda System terminology, it has several disadvantages, including difficulties in organizing quality assurance statistics. This, however, may interfere with the management approach based on current ASCCP guidelines [6,22-25]. The current recommendation is to refer both LSIL and ASC-H cases to colposcopy, but LSIL-H cases have a significantly lower chance of negative results (Table 1), and so relatively aggressive follow up steps may be indicated at later stages of management. Another challenge is difficulty in verifying the risk of progression to high grade dysplasia for two separate interpretations.

HPV DNA testing has been suggested to be a simple alternative with sensitivity and negative predictive value approaching 100% for detecting HSIL [26-29]. The role of HPV testing in primary screening of cervical cancer currently has been effective in the ASCUS category. Its role in other categories is evolving. It has also been reported to be helpful in ASC-H cases [27]. However, in LSIL-H, most of the cases are expected to be positive for HPV testing and so its role may be limited. In the current study, coincidental observation of HPV test results were available in 34 out of total 88 LSIL-H cases. HPV testing was positive for high risk HPV in 94% (32 out of 34). Cervical biopsy in two negative cases showed mild dysplasia with HPV in one (negative for both low-risk and high-risk HPV) and high grade dysplasia- not graded in the other (negative for high-risk HPV but positive for low-risk HPV). These findings, although based on small numbers, suggest that HPV testing is not a useful ancillary test in the management of LSIL-H.

Other possible ancillary tests include ProExC [30,31] and p16^INK4A ^(p16) [19,28,29,32-35]. A few have reported role of ProExC with Topoisomerase II alpha (TOP2A) and minichromosome maintenance protein 2 (MCM2) in detection of cervical high-grade squamous intraepithelial lesions from cytologic samples [30,31]. The current literature supports the role of p16 in squamous dysplasia [19,28,29,32-35]. The results of p16 on cell block sections of cervical cytology specimens [36] can be interpreted more objectively. The interpretation of the specific nuclear immunoreactivity for p16 is consistent with HPV related dysplasia. Nonspecific cytoplasmic staining may be present in surgical pathology and cell block sections, but this does not interfere with the evaluation of nuclear immunoreactivity. However, in cytology smears, this nonspecific cytoplasmic staining interferes with evaluation of nuclear immunoreactivity with certainty [37,38]. Initial observations suggest that p16 may play a significant role as ancillary test [19,32,37]. The role of these molecular markers is evolving and may help in evaluation of LSIL-H in future.

In conclusion, although LSIL-H as interpretation category is not a unique biologic entity, it correlates with increased risk of high grade dysplasia on biopsy. A pattern of biopsy results, intermediate between LSIL and ASC-H but distinct from HSIL, justifies LSIL-H as a separate group for optimal clinical management (Figure 5) with possible application of molecular events such as p16 in the future.

## List of abbreviations

ASC-H, Atypical squamous cells Cannot exclude high-grade intraepithelial lesion; ASCCP, American Society for Colposcopy and Cervical Pathology; ASCUS, Atypical squamous cells of undetermined significance; Bethesda 2001, 2001 Bethesda System terminology; CIN, cervical intraepithelial neoplasia; HPV, human papilloma virus; HPVT, HPV DNA testing; HSIL, high-grade squamous intraepithelial lesion; LSIL, low-grade squamous intraepithelial lesion; LSIL-H, low-grade squamous intraepithelial lesion, cannot exclude high-grade intraepithelial lesion; ND, negative for dysplasia; PPV, Positive predictive value; SAPK cells, small atypical parakeratotic cells.

## Competing interests

The author(s) declare that they have no competing interests.

## Authors' contributions

**VS**, Conceptual organization as senior author, cytological-histological evaluation, data analysis, executing IRB process, and writing the manuscript.

**NK**, Cytopathology fellow, organized IRB process, collected all the data, and review of manuscript.

**RN **&**GB**: Data analysis, review of manuscript, and analyze management algorithm.
